# Visible Epiglottis in Children

**DOI:** 10.5005/jp-journals-10005-1271

**Published:** 2015-02-09

**Authors:** Farooque Jamaluddin Ahmed, Andrá Luis Shinohara, Salete Moura Bonifécio da Silva, Jesus Carlos Andreo, Antonio de Castro Rodrigues

**Affiliations:** Ex-PhD Student, Department of Biological Sciences-Anatomy, Bauru School of Dentistry, University of São Paulo, São Paulo, Brazil; Ex-PhD Student, Department of Biological Sciences-Anatomy, Bauru School of Dentistry, University of São Paulo, São Paulo, Brazil; Associate Professor, Department of Odontopediatrics, Orthodontics and Collective Health, Bauru School of Dentistry, University of São Paulo São Paulo, Brazil; Associate Professor, Department of Biological Sciences-Anatomy, Bauru School of Dentistry, University of São Paulo, São Paulo, Brazil; Professor, Department of Biological Sciences-Anatomy, Bauru School of Dentistry, University of São Paulo, São Paulo, Brazil

**Keywords:** Epiglottis, Larynx, High-rising epiglottis.

## Abstract

Visible epiglottis is a rare anatomical variant which is usually asymptomatic without the need of any medical or surgical intervention. It is most commonly seen in children but there are some reports of its prevalence in adults too. Cases of visible epiglottis seem to be unfamiliar among dental professionals. In this report, we have attempted to present this anatomical variant of epiglottis in the feld of dentistry by describing a case of an 8-year-old girl who presented to the department of pediatric dentistry for normal dental check-up unaware of the existence of the visible epiglottis.

**How to cite this article:** Ahmed FJ, Shinohara AL, da Silva SMB, Andreo JC, de Castro Rodrigues A. Visible Epiglottis in Children. Int J Clin Pediatr Dent 2014;7(3):223-224.

## INTRODUCTION

The Epiglottis is the highest point of the Larynx, which forms the upper portion of the Air Passage. Its main function is to prevent the entry of food into the lungs by closing the trachea (windpipe). The epiglottis along with the aryepiglottic fold helps in directing the water and food toward the upper esophagus.

Embryologically the epiglottis is derived from third and fourth brachial arches.^1^ Congenital anomalies associated with epiglottis are very rare. Hypoplastic epiglottis, rudimentary epiglottis and bifd epiglottis are some of the congenital malformations reported in the literature.^2^ Our thorough search of the literature revealed very few reported cases of visible epiglottis. It has been also called as ‘high-rising epiglottis’ by some other clinicians.^2,3^ Literature does not provide much information about this anatomical variation. Otherwise some authors claim for the importance of the epiglottis anatomy and preepiglottic space in relation to spread of carcinoma of the larynx.^4,5^ This led us to report this benign yet an anatomical variant in the appearance of epiglottis. Unlike bifd epiglottis, visible epiglottis has not been associated with any specific syndrome. Normally, visible epiglottis does not present any physiological distress except in few cases where the patient might complain of difficulty in breathing.

**Fig. 1:**
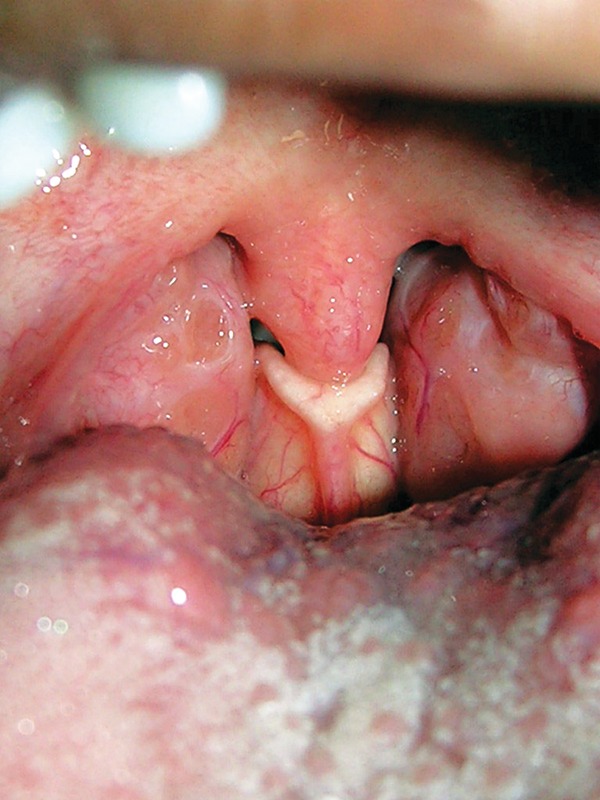
Clinical photograph showing the free border of epiglottis touching the uvula of the patient

## CASE REPORT

An 8-year-old girl visited the Department of Pediatric Dentistry, USP, Bauru-SP, Brazil for a regular oral checkup. While performing oral examination, the dentist came across an unusual anatomical structure located posterior to the tongue. Unaware of its significance, the dentist approached the department of Anatomy, USP for consultation. After a thorough clinical examination of the oral cavity which showed the epiglottis touching the uvula (Fig. 1) and with the help of the literature, a diagnosis of visible epiglottis was made. Knowing the benign feature of such anatomical variant unless associated with any sort of distress, the patient and the parents were reassured of its innocuous nature and the patient was sent back after necessary dental treatment.

## DISCUSSION

In literature, visible epiglottis has mostly been associated with children.^2,3^ However, it is also seen in the adults in certain cases.^6^ Visible epiglottis has been noted during pharyngeal examination for intubation by various clinicians. Ezri et al in the year 1998 added a new class zero to the Mallampati grading system for predicting the degree of difficulty in laryngeal exposure.^7^ Cases in which epiglottis was visible on opening of mouth during laryngoscopy was included in this class zero.^8^ In a further study carried out by Ezri et al (2001) (class zero airway had an incidence of 1.18% in adults. This rate is much higher in children as observed by Raghavendran and Vas where they report 6 such cases out of 100 examined in children aged between 6 and 10 years. Interestingly most of the cases reported involved female subjects suggesting a sexual predilection toward female of this anatomical variant.^6,9^

Knowledge of ‘visible epiglottis’ or the high rising epiglottis is important as it might cause unnecessary panic in parents and sometimes even among the dental professionals. Even though it is not very commonly witnessed, a lack of knowledge of such cases can become a cause of concern for the dental professionals as seen in this particular case. A lot of queries from the parents have been made in internet regarding visible epiglottis.^2^ General awareness of this anatomical variant will reduce the anxiety level among the population. No treatment has been suggested for cases without accompanying any sorts of distress in the patient. If a dentist comes across such cases and the patient complains about any discomfort, they should be referred to an otolaryngologi-cal consultation.

## CONCLUSION

Knowledge of ‘visible epiglottis’ or the high rising epiglottis is important as it might cause unnecessary panic in parents and sometimes even among the dental professionals.
